# Genetic characteristics of spouse selection based on short tandem repeats in DNA and lunula count on fingertip

**DOI:** 10.1186/s41021-023-00281-6

**Published:** 2023-10-20

**Authors:** Qi Xia, Ullah Anwar, Yu Weijian, Wang Yingshuai, Liu Hui

**Affiliations:** College of Medical Laboratory, Dalian Medical University, Dalian, 116044 China

**Keywords:** STR, Lunula, Nail matrix, Heredity, Spouse selection, Genetic marker

## Abstract

**Objective:**

The aim of this study was to assess the correlation of spouse selection with **s**hort tandem repeats (STRs) in DNA and with the number of fingertip lunulae to investigate the role of heredity in spouse selection.

**Methods:**

We randomly selected a total of 286 couples (husband and wife) as a couple group while 200 paired subjects (a man randomly matched with a woman as a pair of subjects) were selected as a non-spouse group for DNA typing, and to investigate lunulae in spouse selection, a total of 554 couples were selected as a couple group and 500 pairs of subjects were selected as a control group.

**Results:**

A significant difference of STR matching number (a large value implies a higher genetic similarity) between spouse group and non-spouse group were observed (12.3 ± 2.7 vs. 11.8 ± 2.6; p < 0.05). A significant difference of the lunula matching number (difference of lunula counts between a paired subjects, a lower value implies a higher genetic similarity) between two groups were also observed for the lunula counts (1.55 ± 1.88 vs. 3.53 ± 2.40; p < 0.01).

**Conclusion:**

Significant and unprecedented relationships were found between the couples and polymorphic STRs, and between spouse selection and lunula counts. Polymorphic STRs and fingertip lunulae counts provide an initial insight into the potentially important contributions that genetic characteristics may play a key role in spouse selection.

## Introduction

Selecting a life partner is a complex and deeply personal decision. While cultural, social, and personal preferences often play a significant role, an emerging perspective suggests that biological factors and genetic characteristics may also influence spouse selection. Biological factors refer to the innate qualities and characteristics that individuals possess. These factors encompass physical attributes such as height of the body and appearance, personality traits, and even hormonal influences. Evolutionary psychology suggests that certain physical features or qualities may signal fertility, health, and reproductive fitness, triggering subconscious preferences in mate selection [1]. Beyond physical attraction, genetic compatibility also factors into spouse selection.Genetic traits can influence various aspects of life, including disease susceptibilities, temperament, and cognitive abilities. Finding a partner with compatible genetic traits can potentially lead to healthier and more resilient offspring, as it reduces the risk of genetic disorders and enhances overall genetic fitness [2, 3]. It is well known that these behavioural and biologic characteristics have a genetic basis. However, there has been limited available information to demonstrate this hypothesis when evaluating spouse selection [4].

STRs (short tandem repeats),also known as micro-satellite DNA [5]. The core sequence consists of 2-6 bp head-to-tail tandem repeats that count for approximately 3% of the human genome usually ranging from 4 to 60 repeats. In biological research, STRs are widely used because of their high mutation rates and polymorphism [6, 7].When STRs are analyzed for identification purpose, the number of repeat units is reported to be highly variable among individuals, which present high discrimination, STRs are found non coding in nature and it’s a widely accepted notion, therefore, not involved in gene expression [8, 9]. However, more evidence has been reported that in various mechanisms non coding DNA sequence such as STRs are may be implicated in the gene regulation, so being associated with phenotype [10, 11]. Investigating genetic polymorphism of 19 STR loci including *FGA, D13S317, TPOX, TH01, D3S1358, D18S51, D19S433, D21S11, D8S1179, D5S818, D2S1338, D6S1043, D7S820, CSFIO, D16S539, D12S391, vWA, Penta E and Penta D* is an important method in human genetics. It’s currently used by forensic laboratories and the U.S. national Combined DNA Indexing System (CODIS) [12, 13]. This procedure can be performed at semi automated conditions as per international standards as a reliable assay with commercial multiplex kits, and then the results are evaluated by supporting software; therefore, errors due to individual judgements and artificial operations are maximally reduced [14]. This procedure was therefore chosen for this study as a reliable genotyping assay. We have an assumption that there is an extensive range of spouse-associated genes in the human genome; therefore, we selected 19 STR loci to screen for spouse-related genes.

**Fig. 1:**
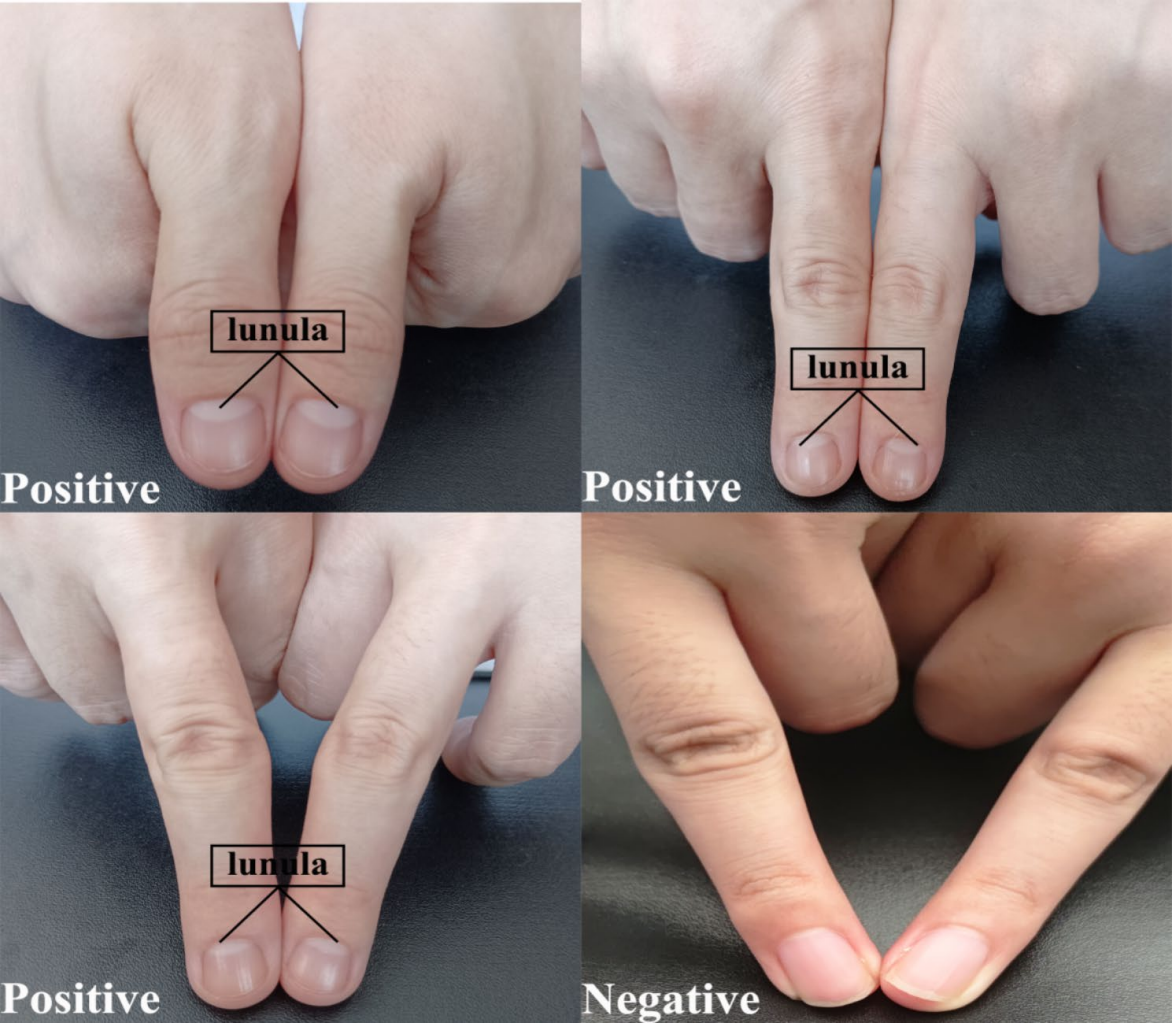
Lunula of fingertips

The lunula is the moon-shaped white-coloured region at the base of a fingernail that can be observed on some digits (Fig. 1). In fact, the lunula is the visible part of the root of the nail and seem whiter than the rest of the nail because of the tissue arrangement in the area where a thickened underlying stratum basale obscures the underlying dermal blood vessels [15]. This structure appears during embryonic development. This feature is most noticeable on the thumb; however, it is not necessarily visible on every individual. Therefore, the counts among the ten fingers are typically different for each individual and could be considered an important model for research on genetic biomarkers [16, 17]. Several reports have indicated that the fingertip lunula may relate to particular diseases [18, 19]. We have also hypothesized that such a feature could be a key genetic marker related to spouse selection.

Interestingly, significant and unprecedented relationships were found between the couples (husband and wife) and polymorphic STRs and between spouse selection and lunula count. To the best of our knowledge, this phenomenon has not been reported before.

## Methods

### Subject

We randomly selected a total of 286 couples (husband and wife) as the couple group and 200 pair of subjects (a man randomly matched with a woman as a pair of subjects) as the non-spouse group for typing DNA.

We also randomly selected a total of 554 couples (husband and wife) as the couple group and 500 pairs of subjects (a man randomly matched with a woman as a pair of subjects) as the non-spouse group for observing the role of lunula in spouse selection. The lunulae data were obtained by self-report surveys or telephone interviews.

### STR amplification

Genomic DNA was extracted from all blood samples by using Automated Nucleic Acid Extractor (Magcore, Taipei, Taiwan). Polymerase chain reaction (PCR) was carried out by GoldenEye™ 20 A Kit (Beijing PeopleSpot Inc., Beijing, China). A total of 19 STR loci (*CSF1PO, D13S317, D12S391, D7S820, D16S539, D18S51, D21S11, D2S1338, D3S1358, D19S433, D5S818, D6S1043, D8S1179, Penta D, Penta E,FGA, TPOX ,TH01 and vWA*)were amplified. After amplification, PCR products were loaded into an ABI 3130-Avant Genetic Analyzer (Applied Biosystems, Foster City, CA) for capillary electrophoresis and the collected data were automatically analyzed with GeneMapper® ID Software v3.2 (Applied Biosystems, Foster City, CA). The 19 STR loci that were amplified as shown in the Table 1.

**Table 1:** Amplification parameters for STR loci

Locus	GenBank accession number	PCR product size (bp)	Repeated sequences	Chromosomal location
vWA	M25858	121–183	(TCTA)_n_(TCTA)_n_	12p13.31
CSF1PO	X14720	313–357	(AGAT)_n_	5q33.2
TPOX	M68651	262–314	(GAAT)_n_	2p15.3
FGA	M64982	278–434	(TTTC)_n_	4q28
TH01	D00269	92–136	(TCAT)_n_	11p15.5
D3S1358	NT-005997	106–154	(TCTA)_n_	3p21.31
D13S317	G09017	162–210	(TATC)_n_	13q31.1
D8S1179	G08710	203–255	(TCTA)_n_	8q24.13
D5S818	G08446	138–186	(AGAT)_n_	5q22
D16S539	G07925	257–305	(GATA)_n_	16q24.1
D7S820	G20012	208–252	(GATA)_n_	7q21.11
D2S1338	G08202	211–271	(TGCC)_n_	2q35
D19S433	G08036	77–135	(AAGG)_n_	19q12
D21S11	M84567	194–268	(TCTA)_n_	21q21.1
D12S391	G08921	145–201	(AGAT)_n_(AGAC)_n_	12p13.2
D6S1043	G08539	377–441	(TCTA)_n_	6q15
D18S51	L18333	280–367	(GAAA)_n_	18q21.33
Penta E	NT-010274	316–431	(AAAGA)_n_	15q26.2
Penta D	AP001752.1	363–446	(AAAGA)_n_	21q22.3

The number in the pane represents the number of core unit repeats along with the name of the STR allele

### Data analysis

A quantitative numerical method was used to analyse the matching of each STR locus according to the literature [20].The matching values of the two alleles at each locus were scored from 0 to 2 as showed in Table 2. At each gene locus, the sum of the STR matching numbers in both the spouse group and the non-spouse group were calculated as the matching value; a larger value implies a higher genetic similarity. The differences in the sum of the matching numbers for the alleles at 19 STR loci in the two groups were analyzed using *t* tests.

**Table 2:** The matching values assigned for the two alleles at one locus

Loci		Chromosome	Score
Subject 1	Subject 2		
a	c	1	0
b	d	2	
a	a	1	1
b	c	2	
a	a	1	1
a	c	2	
a	a	1	2
b	b	2	

Different alleles are representing by a, b, c and dat a particular locus

Lunula counts for ten fingers were obtained for each subject. The differences in lunula counts between a couple and that between a randomized pair of subjects were respectively calculated as a matching value; a lower value implies a higher genetic similarity. The differences in the sums of matching values in the two groups were analyzed by using *t* tests.

### Statistical analysis

The results were analyzed using SPSS software version 20 (Chicago, IL, USA). Differences of matching values between two groups were compared using the independent-samples *t* test. The constituent ratios for each count were examined using the chi-square test.*P*-value < 0.05 (using a two-tailed test) was considered as statistically significant difference.

## Results

**Fig. 2:**
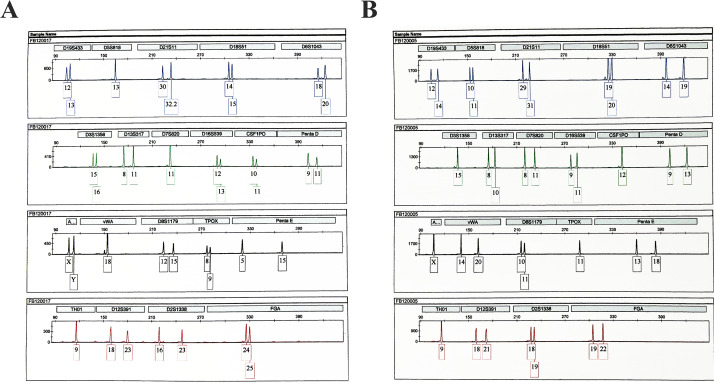
STRs graphical results (the number in the pane represents the number of core unit repeats along with the name of the STR allele). **A**: Sample 1 (D19S433: 12, 13; D5S818: 13, 13; D21S11: 30, 32.2; D18S51: 14, 15; D6S1043: 18, 20; D3S1358: 15, 16; D13S317: 8, 11; D7S820: 11, 11; D16S539: 12, 13; CSF1PO: 10, 11; Penta D: 9, 11; vWA: 18, 18; D8S1179: 12, 15; TPOX: 8, 9; Penta E: 5, 15; TH01: 9, 9; D12S391: 18, 23; D2S1338: 16, 23; FGA: 24, 25). **B**: Sample 2 (D19S433: 12, 14; D5S818: 10, 11; D21S11: 29, 31; D18S51: 19, 20; D6S1043: 14, 19; D3S1358: 15, 15; D13S317: 8, 10; D7S820: 8, 11; D16S539: 9, 11; CSF1PO: 12, 12; Penta D: 9, 13; vWA: 14, 20; D8S1179: 10, 11; TPOX: 11, 11; Penta E: 13, 18; TH01: 9, 9; D12S391: 18, 21; D2S1338: 18, 19; FGA: 19, 22)

The raw data of alleles in the 19 STR loci was obtained with Genescan Analysis software. The STR typing profiles were automatically obtained for each sample. The alleles were consistent with the theory, as shown in Fig. 2.

Quantitative numerical method was used to analyze matching at each STR locus, as shown in the Table 3. STR matching number (a larger value implies a higher genetic similarity) was 12.3 ± 2.7 in spouse group and 11.8 ± 2.6 in non-spouse group. Using a cut off of *p* < 0.05, a significant difference of matching number between the two groups were observed (*p* = 0.040).

**Table 3:** Similarity of 19 STR loci analyzed by using a quantitative numerical method

Groups	N	Matching value*	P-value
Husband and wife	286	12.3 ± 2.7	0.040
Pair matched randomly	200	11.8 ± 2.6	

*Number of same STR alleles between a paired subjects (a larger value implies a higher genetic similarity)

The initial data composed of the lunula counts are listed in Table 4.These results indicate that the constituent ratios for each count were similar among the husband, wife and random population (p > 0.05).

**Table 4:** Initial data collection of lunula counts in husband, wife and random population

Lunula count	Husband		Wife		Random population	
	n	%	n	%	n	%
0	23	4.15	23	4.15	44	4.40
1	3	0.54	3	0.54	26	2.60
2	50	9.03	60	10.83	158	15.80
3	29	5.23	29	5.23	64	6.40
4	79	14.26	91	16.43	112	11.20
5	43	7.76	53	9.57	85	8.50
6	109	19.68	89	16.06	123	12.30
7	55	9.93	55	9.93	60	6.00
8	62	11.19	64	11.55	132	13.20
9	18	3.25	24	4.33	57	5.70
10	83	14.98	63	11.37	139	13.90
Total	554	100	554	100	1000	100

n = number of individuals

The matching values for the lunula counts(difference of lunula counts between a paired subjects, a lower value implies a higher genetic similarity) were also analyzed in the two groups as shown in the Table 5. The matching values for the lunula counts were 1.55 ± 1.88 in spouse group and 3.53 ± 2.40 in non-spouse group. Significant differences of matching values between the two groups were also observed (*p* < 0.001).

**Table 5:** Similarity of lunula count analyzed by using a matching value

Groups	N	Matching value*	P-value
Husband and wife	554	1.55 ± 1.88	< 0.001
Pair matched randomly	500	3.53 ± 2.40	

*Difference of lunula counts between a paired subjects (a lower value implies a higher genetic similarity)

## Discussion

As we know, mate choice involves biological and sociological factors and is not random. Existing research suggests that mate’s selection may be correlated with MHC (major histocompatibility complex) [21–23]. According to sexual selection theory, choosing mates who have different MHC genes may help preserve genetic in animals population by preventing inbreeding or boosting future offspring resistance to disease. However, there is still no evidence that STRs have a correlation to spouse selection.

STRs consist of tandemly repeated DNA units ranging from two to six nucleotides (Table 1). They are one of DNA fingerprinting with highly polymorphic variable number of tandem repeats. STRs are also the most powerful genetic tools in human and other species on molecular marker-based analysis, such as the genetic diversity assessment of rice germplasm using molecular markers, DNA markers RAPD and SSR to assess the genetic diversity of rice germplasm, genetic diversity and population structure of wheat landraces, molecular marker-based analysis of genetic diversity and population structure of tomato landraces, DNA fingerprinting and genetic diversity analysis of medicinal plant species in the genus Curcuma and so on [24–27]. In this study, we wondered whether or not STRs are linked with spouse selection, and conducted a series of experiments to explore it.

In the present study, we examined 19 STR loci disseminated across various chromosomes. As per international standard half-automated procedure was used, using commercial multiplex kits and reliable assays. To minimize bias and reduce operating errors, all data were analyzed with standard software packages. In theory, at 19 STR loci the alleles can be distributed randomly. Thus, STRs polymorphisms and other genes, such as HLA polymorphisms, are associated to different diseases very closely [28–31]. At these STR loci, an imbalance in the allele distribution may be seen in the couples. Our results showed that the numbers of same STR alleles between spouses were more than that between randomly matched population, and lunula counts were a strong similarity between husband and wife and support the above-mentioned hypothesis. We studied in the couple groups that in STR alleles, the matching values were significantly greater than in the random population, indicating that spouse selection may have a genetic basis.

Traditionally, when choosing a spouse, these external genetic factors, such as height and weight can affect the spouse selection, which can be attributed mainly to the role of social factors. The lunula counts of the fingertips were not used as an objective indicator, which can be attributed mainly to the role of biological factors. The ermatoglyphics of human fingertips as biomarkers have already been reported in spouse selection [32].In this study, it was found that the lunula counts between the couples had a very high similarity, when studying the genetic relationship of the lunula count of the fingertips in the spouse selection. Moreover, the lunula count distributions of the mates were different from the random population.

In the present study, the fingertip lunula counts were obtained from 554 randomly selected couples and 1000 individuals from the random population. We found that the constituent ratios for each count were distinctly similar among the husband, wife, and random populations, indicating the collection of reliable data. In theory, the lunula count of a spouse is expected to be independent of that of their partner, resulting no inherent relationship in spouse selection. Nevertheless, our results exhibited an exceptional distribution between spouses compared with the randomly matched population, which was contrary to the common random independent assortment of couples. Therefore, it is important to approach the discussion of biological factors and genetic characteristics with sensitivity, respecting individual autonomy and diverse cultural perspectives. Further research is necessary to fully comprehend the intricacies of how biological factors and genetic characteristics play a role in spouse selection and to establish ethical guidelines for any potential applications.

## Conclusion

Upon extensive inspection of our data, we observed that numbers of same STR alleles between spouses (matching value) were more than that between randomly matched population, and lunula counts were a strong similarity between husband and wife.The cross-validation of the above two mentioned analytical results showed a notable contribution to spouse selection. The idea that biological factors and genetic characteristics influence spouse selection adds depth to our understanding of human relationships. As technology and scientific knowledge continue to evolve, it is important to approach this topic with an open mind, seeking a balance between scientific evidence, personal agency, and cultural context to ensure that discussions related to spouse selection remain respectful and inclusive for healthier and more resilient offspring.

## Data Availability

No additional data are available all relevant data are provided with in the manuscript.

## Declarations

### Data transparency

Authors will ensure data transparency.

## Abbreviations

MHC: histocompatibility complex
PCR: polymerase chain reaction
STR: short tandem repeats
